# New Biochemical Insights into RIT GTPases Regulation and Membrane Interactions

**DOI:** 10.3390/cells15171567

**Published:** 2026-08-28

**Authors:** Amin Mirzaiebadizi, Farhad Bazgir, Niloufar Mosaddeghzadeh, Silke Pudewell, Neda S. Kazemein Jasemi, Radovan Dvorsky, Mohammad R. Ahmadian

**Affiliations:** Institute of Biochemistry and Molecular Biology II, Medical Faculty, Heinrich Heine University Düsseldorf, Universitätsstrasse 1, Building 22.03, 40225 Düsseldorf, Germany; amin.mirzaiebadizi@hhu.de (A.M.); farhad.bazgir@cardioscience.uni-heidelberg.de (F.B.); niloufar.mosaddeghzadeh@uk-koeln.de (N.M.); silke.pudewell@hhu.de (S.P.); neda.kazemi22@gmail.com (N.S.K.J.); radovan.dvorsky@gmail.com (R.D.)

**Keywords:** RIT1, RIT2, RAS superfamily, small GTPases, lipid interactions, phosphoinositide binding, Galectin-3, LZTR1, RASopathies

## Abstract

**Highlights:**

**What are the main findings?**

- RIT1 exhibits different nucleotide cycling kinetics compared to classical RAS.
- RIT1 and RIT2 bind to membrane lipids via a basic C-terminal extension.
- The KRLK motif mediates RIT binding to phosphoinositides.
- Galectin-3 and LZTR1 reduce RIT-liposome association in a reconstituted system.

**Abstract:**

Both RIT1 and RIT2 are members of the RAS superfamily of small GTPases, which regulate various cellular processes. RIT1 is widely expressed, whereas RIT2 is primarily found in neuronal tissues. Dysregulation of these proteins has been associated with several human diseases, including Noonan syndrome, cancer, Parkinson’s disease, autism, and schizophrenia. Although RIT1 and RIT2 are often compared to classical RAS proteins, they exhibit distinct regulatory and biochemical properties. Here, we demonstrate that RIT1 differs from classical RAS in GTPase cycling. Unlike classical RAS proteins, RIT1 did not respond to SOS1-mediated nucleotide exchange or p120GAP-stimulated GTP hydrolysis under cell-free conditions. These results imply that RIT1 may depend on regulatory mechanisms that differ from those of classical RAS proteins. However, the relevant physiological regulators remain unknown. Disease-associated RIT1 mutations cluster around the P-loop and Switch II regions. In this transient overexpression screening system, however, these mutations had only a modest effect on the canonical MAPK, PI3K/AKT, and JNK signaling pathways in HEK293T overexpression experiments. This suggests the existence of additional context-specific effectors and regulatory factors. We demonstrate that RIT1 and RIT2 interact with membrane lipids via a basic C-terminal extension. The KRLK-containing region contributes to the binding of phosphatidylserine and phosphoinositides. Charge-reversal mutations disrupt lipid interactions and liposome binding, supporting the functional importance of this region. In a reconstituted liposome system, galectin-3 and LZTR1, but not galectin-1, reduced the interaction of GDP-loaded RIT1 and RIT2 with liposomes. These results suggest that accessory proteins may influence RIT membrane interactions. However, their cellular relevance requires further validation. Together, our findings provide biochemical insights into RIT GTPase regulation and its interactions with membrane lipids under cell-free conditions.

## 1. Introduction

Small GTPases of the RAS family act as molecular switches within cells, cycling between an active GTP-bound state and an inactive GDP-bound state [1]. These proteins mediate intracellular responses to extracellular signals transmitted via receptors and adaptor proteins, ultimately influencing diverse downstream effectors. Through this signaling relay, RAS proteins regulate essential cellular processes, including gene expression, metabolism, cell cycle progression, proliferation, survival, and differentiation. Somatic or germline mutations in RAS family genes or their regulators are frequently associated with cancer and developmental disorders collectively termed RASopathies [2]. These disorders are usually caused by germline mutations in components of the highly conserved RAS–MAPK pathway. Mild hyperactivation of this pathway is considered the main pathogenic mechanism underlying syndromes with similar symptoms, including Noonan syndrome (NS), Costello syndrome (CS), and cardio-facio-cutaneous syndrome (CFCS) [3].

**Figure 1:**
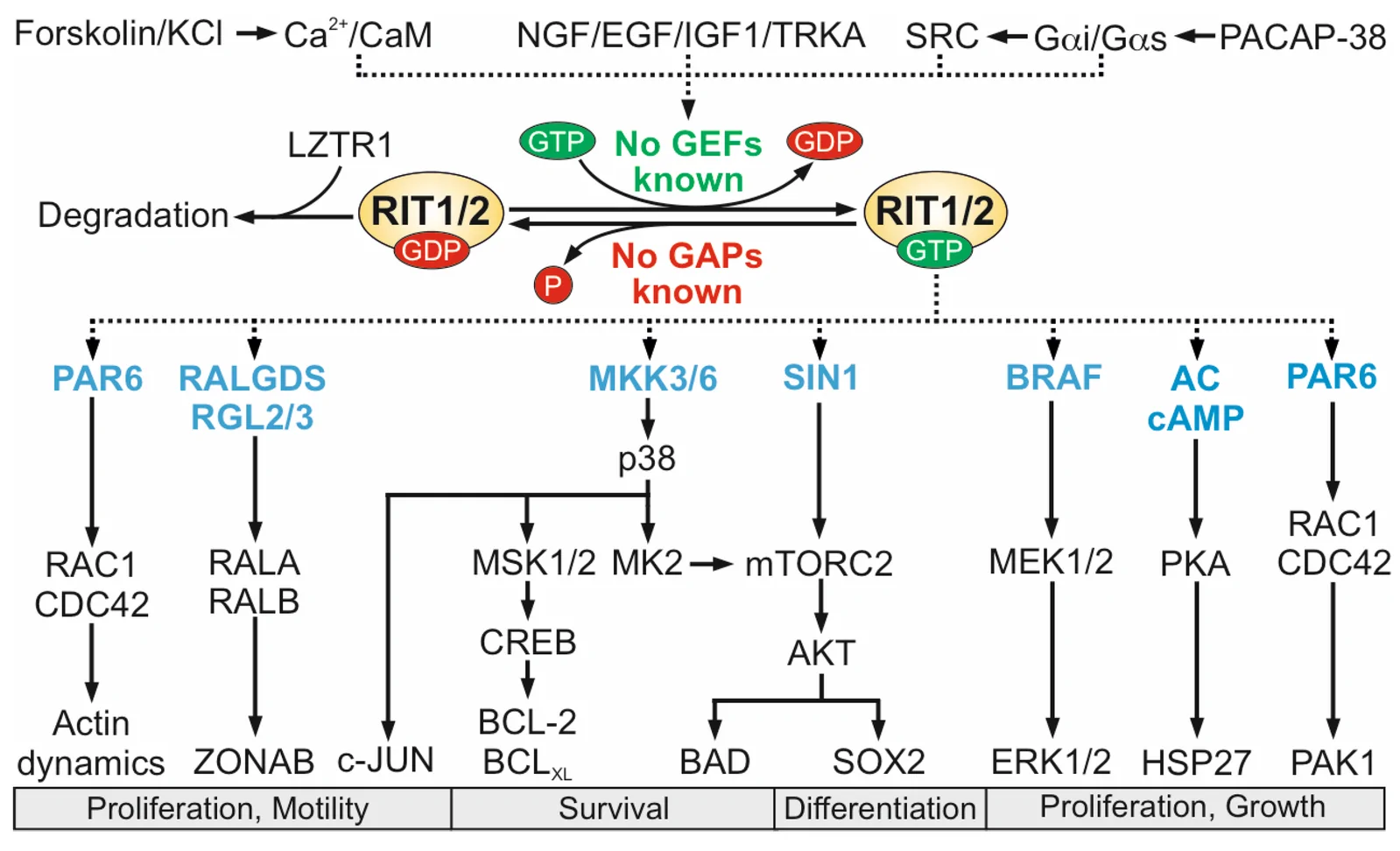
Putative RIT1 and RIT2 signaling pathways. RIT1 and RIT2 function as molecular switches by cycling between an inactive GDP-bound (off) state and an active GTP-bound (on) state. Their activation and inactivation are believed to involve guanine nucleotide exchange factors (GEFs; green) and GTPase-activating proteins (GAPs; red). However, no regulators of RIT1 and RIT2 have yet been identified. Several upstream signals have been proposed to influence RIT1 and RIT2 signaling via proposed effector proteins (shown in blue). The proposed interactions are highlighted by dashed arrows, while the findings are indicated by solid arrows. These signals might contribute to various cellular processes. Further details are provided in the main text. This figure was created using CorelDRAW 2020.

The best-characterized RAS proteins are HRAS, NRAS, and KRAS4B. While these proteins exhibit overlapping functions, they differ in their expression patterns, regulatory partners, and subcellular localization. This underscores their functional specificity and partial redundancy [4,5]. Most RAS proteins undergo post-translational modification at a conserved C-terminal CAAX motif (C for cysteine, A for an aliphatic amino acid, and X for any amino acid), which allows them to be anchored to the membrane through prenylation. However, RIT1 (also known as RIT, RIBB, ROC1) and RIT2 (also known as RIN, ROC2) lack this motif. Instead, they associate with membranes through a distinct mechanism that relies on their positively charged C-terminal extensions [1,6]. The functions of many other RAS family members also remain incompletely characterized. RIT1, which is ubiquitously expressed, and its neuron-specific paralog RIT2 were first identified in the mouse retina in 1996 as a distinct RAS-related subfamily [7]. Since then, various animal and cellular models have revealed their involvement in regulating cell survival, proliferation, differentiation, and morphogenesis (Figure 1) [1,8]. RIT1 and RIT2 also contribute to neurogenesis, neurite outgrowth, and neuronal branching [8]. Studies have linked RIT1 and RIT2 signaling to various malignancies, neurodegenerative disorders (e.g., Parkinson’s disease), and neurodevelopmental phenotypes (e.g., autism, schizophrenia, NS and CS) [8,9,10,11,12,13,14]. For example, hypoxia-induced HIF-1α upregulates RIT1 in hepatocellular carcinoma, thereby promoting tumor migration, invasion, and drug resistance [15].

Although the upstream signals that activate RIT1 and RIT2 appear to be similar, the mechanisms that regulate them remain unclear. Various extracellular stimuli, including EGF, NGF, IGF-1, and PACAP38, activate RIT1 and RIT2 in both neuronal and non-neuronal contexts (Figure 1) [16,17,18]. Thus far, no guanine nucleotide exchange factors (GEFs) or GTPase-activating proteins (GAPs) have been identified that directly regulate RIT1 or RIT2. EPAC1, a cAMP-dependent RAPGEF, has been shown to link PACAP38 signaling to RIT1 activation; however, it does not exhibit direct catalytic activity toward RIT1 [19]. Subsequent studies revealed that EPAC1 activates SRC-dependent TRKA signaling, which may stimulate RIT1 via SOS1 or SOS2 [20]. Neither EPAC1 nor SOS1 has been shown to directly activate RIT1 in cell-free assays. Thus, the identity of its direct upstream regulators remains unclear [20].

Effector binding and downstream pathway activation are central to RAS-family signaling. Several pathways have been proposed for RIT1 and RIT2 based on their potential interactions with known RAS effectors (Figure 1) [1,8,9]. RIT1 has been suggested to interact with RALGDS family members, such as RALGDS and RGL3, which could potentially link it to RAL signaling [20]. A glutathione S-transferase (GST)-tagged RGL3 RAS-association domain (residues 610–709) is commonly used to detect GTP-bound RIT1 in cell lysates. RIT1 also activates a p38 MAPK-dependent stress resistance pathway that promotes cell survival, a function not typically shared by other RAS family members [21]. Additionally, RIT1 activates the p38-MSK1-CREB pathway to induce anti-apoptotic proteins such as BCL-2 and BCL-XL, and drives c-JUN transcription via the MKK3/MKK6-p38γ axis (Figure 1) [22]. Through the p38-MK2-HSP27 cascade, RIT1 also promotes AKT activation and BAD phosphorylation, thereby suppressing ROS-induced apoptosis [21]. In neuronal contexts, RIT1 has been implicated in modulating NGF-induced MEK-ERK and p38 signaling pathways, possibly via crosstalk with BRAF, while operating independently of PI3K-AKT signaling [16,23,24]. However, the precise structural and mechanistic connections governing RIT1-dependent ERK1/2 activation remain to be fully elucidated. RIT1 regulates the proliferation and differentiation of adult neural progenitors via the SIN1–mTORC2–AKT axis, which mediates the phosphorylation of SOX2, a key stem cell transcription factor [25]. RIT1 binds to SIN1 and may regulate mTORC2-mediated AKT phosphorylation [26]. RIT2 participates in PACAP38–Gαs–SRC signaling and controls neuronal differentiation through RAC1- and CDC42-dependent cytoskeletal remodeling [27]. It regulates RAC1 and CDC42 activation via PAR6, thereby influencing neurite outgrowth [28]. RIT1 modulates the dynamics of the actin cytoskeleton through complexes containing RAC1, CDC42, and PAK1 [28,29,30]. Due to the diversity of downstream pathways, it is reasonable to assume that RIT proteins function through scaffold proteins that can coordinate multiple signaling inputs (Figure 1).

In this study, we examined the biochemical regulatory properties of RIT1 alongside the membrane interaction profiles of both RIT1 and RIT2 under defined cell-free conditions. This analysis aimed to elucidate the differences between these proteins and classical RAS GTPases. Specifically, we examined the following: (1) the intrinsic and extrinsic nucleotide-cycling behavior of RIT1; (2) its responsiveness to the canonical RAS regulators SOS1 and p120GAP; (3) the membrane lipid-binding selectivities of RIT1 and RIT2; and (4) the regulatory effects of galectin-3 (GAL3) and LZTR1 on RIT1- and RIT2-liposome interactions in a reconstituted system. These complementary biochemical approaches together provide novel insights into the regulatory dynamics and membrane-association properties of RIT GTPases. While these cell-free data establish a rigorous biochemical framework, their subsequent validation in physiologically relevant cellular systems is also necessary.

## 2. Materials and Methods

### 2.1. Constructs

pGEX-4T-1 vectors encoding N-terminal GST fusion proteins were used to express the following proteins in Escherichia coli BL21 (Rosetta): full-length human RIT1 (accession number or AccN: Q92963), full-length human RIT2 (AccN: Q5BJQ5), and the C-terminal extension (Cex) constructs of both proteins. The following RIT1 Cex variants were generated for lipid-binding analysis: RIT1-WT (amino acids or aa 183–219: RRKEKEAVLAMEKKSKPKNSVWKRLKSPF RKKKDSVT), RIT1-M1 (EEKEKEAVLAEK KSKPKNSVWKRLKSPFRKKKDSVT), RIT1-M2 (RRKEKEAVLAEKKSEPENSVWKRLKS PFRKKKDSVT), RIT1-M3 (RRKEKEAVLAMEKKSKPKNSVWEELESPFRKKKDSVT), RIT1-M4 (RRKEKEAVLAMEKKSKP-KNSVWKRLKSPFEEEKDSVT), and RIT1-M5 (EEK EKEAVLAMEKKSEPENSVWEELESPFEEEK DSVT). The RIT2 Cex construct encompassed residues 182–217 (RKKE SMPSLMEKKRKD SLWKKLKGSLKKKRENMT). The mutational design excluded residues that were positively charged and immediately adjacent to negatively charged residues. For lipid-binding controls, the PH domain of human SIN1 (AccN: Q9BPZ7; aa 373–522) and GST alone (AccN: P08515) were cloned into pGEX-4T-1. For bacterial expression, full-length human Galectin-1 (GAL1; AccN: P09382) and Galectin-3 (GAL3; AccN: P17931) were cloned into pET-28a with N-terminal His6 or decahistidine tags, respectively. Full-length human LZTR1 (AccN: Q8N653) was expressed in mammalian cells using pcDNA-3.1 with a C-terminal His6 tag. Additional constructs for GEF, GAP, and effector interaction analysis were cloned into pGEX-4T-1. These constructs included human HRAS (AccN: P01112), the REM-CDC25 domain of human SOS1 (AccN: Q07889; aa 564 to 1045), the GAP domain of p120GAP (AccN: P20936; aa 714–1049), Calmodulin (AccN: P0DP23; aa 1–149), RGL3 RA (AccN: Q3MIN7; aa 612–707), RALGDS RA (AccN: Q12967; aa 777–872), RASSF7 (AccN: Q02833; aa 6–89), RASSF9 (AccN: O75901; aa 25–119), CRAF RBD (AccN: P04049; aa 51–131), CRAF RBD–CRD (AccN: P04049; aa 51–184), ARAF RBD (AccN: P10398; aa 19–91), BRAF RBD–CRD (AccN: P15056; aa 157–280), PI3Kγ RBD (AccN: P42336; aa 169–301), PLC RA (AccN: Q9P212; aa 2130–2240), RIN1 RA (AccN: Q13671; aa 624–716), and PAK1 RBD (AccN: Q13153; aa 57–141). For mammalian expression, HA-tagged wild-type RIT1 and its mutants (K23N, G31R, A57G, Q79L, F82L, M90V, and G95A) were cloned into the pMT2 vector and transfected into HEK293T cells.

### 2.2. Proteins

Adding 0.5 mM IPTG induced protein expression when the OD600 reached 0.6–0.8. The cells were then incubated overnight at 18 °C. The cell pellets were lysed by sonication in a lysis buffer containing 50 mM Tris-HCl (pH 7.5), 300 mM NaCl, 5 mM MgCl_2_, 1 mM DTT, DNase I, lysozyme, Triton X-100, and a protease inhibitor cocktail. The clarified lysates were loaded onto glutathione–Sepharose columns, and the GST fusion proteins were eluted according to standard protocols [31,32]. Where required, GST tags were removed by thrombin cleavage followed by a second round of affinity purification. The purified proteins were then buffer-exchanged into a solution containing 30 mM Tris-HCl or HEPES, 150–500 mM NaCl, 5–10 mM MgCl_2_, 3 mM DTT or β-mercaptoethanol, and 1–5% glycerol. The pH was adjusted to approximately one unit higher or lower than the respective isoelectric point of the respective proteins. For the isolated full-length small GTPases, the same buffer was used plus 0.1 mM GDP. Protein purity was assessed by SDS-PAGE, and aliquots were stored at −80 °C. GAL1 and GAL3 were expressed in *E. coli* BL21 as N-terminal His-tagged proteins. They were purified on Ni-NTA resin and eluted with imidazole-containing buffer. Full-length LZTR1 was expressed in Expi293F mammalian cells as a C-terminal His-tagged protein, as previously described [33]. The cells were transfected at a high density and harvested after three to four days. The cells were lysed in a detergent-containing buffer that was supplemented with protease and phosphatase inhibitors. The soluble fractions were then purified by Ni-NTA affinity chromatography, eluted with imidazole, buffer exchanged, concentrated, snap-frozen in liquid nitrogen, and stored at −80 °C. Nucleotide-free and fluorescently labeled proteins were generated enzymatically at 4 °C using alkaline phosphatase coupled to agarose beads and phosphodiesterase, as described [31,32]. The proteins were subsequently loaded with fluorescent nucleotides, such as mant-GppNHp, mant-dGDP, or TAMRA-GTP. GppNHp is a non-hydrolyzable GTP analog. Excess nucleotides were removed using NAP-5 desalting columns. The nucleotide concentration was determined by high-performance liquid chromatography (HPLC). Protein concentration was determined by the Bradford assay, and protein purity was evaluated by Coomassie-stained SDS-PAGE.

### 2.3. Structural Modeling

We performed structural modeling and sequence analysis to investigate the conserved features of RIT1 and the locations of disease-associated mutations. Amino acid alignments of RIT1, RIT2, HRAS, and KRAS were generated using the ClustalW algorithm within the BioEdit V7.2.5 software suite to identify conserved G motifs (G1–G5) and to map mutational hotspots across these paralogs. We used the refined GDP-bound structure of RIT1 (PDB ID: 4KLZ) to visualize the secondary structure elements, which include α-helices, β-strands, and switch regions I and II. We also examined the clustering of germline and somatic mutations reported in cancers and RASopathies. Mutational residues were displayed on the RIT1 surface using the PyMOL 2.4 molecular viewer, and their spatial proximity to functional motifs was examined. The alignment confirmed that key residues involved in nucleotide binding and hydrolysis (e.g., G31, K34, and S35) are conserved. However, deviations in the switch regions and at specific positions (e.g., F82, which corresponds to Y64 in HRAS) may contribute to the reduced responsiveness of RIT1 to canonical RAS regulators. Comparative surface representations highlighted the distribution of disease-associated mutations and their localization in structurally tolerant regions or near functionally relevant pockets. This provides a framework for interpreting their biochemical effects. All structural figures were rendered in PyMOL and annotated according to the domain architecture derived from the crystallographic model.

### 2.4. Nucleotide Exchange and Binding Assays

The nucleotide exchange and binding kinetics of RIT1 and HRAS were analyzed using a Horiba Fluoromax-4 fluorimeter and an SX20 stopped-flow spectrometer (Applied Photophysics Limited, Leatherhead, UK). Intrinsic and GEF-stimulated reactions were performed in a buffer consisting of 30 mM Tris-HCl (pH 7.5), 10 mM KH_2_PO_4_/K_2_HPO_4_, 5 mM MgCl_2_, and 3 mM DTT at 25 °C. For intrinsic exchange, 0.1 µM mant-dGDP–loaded protein was rapidly mixed with a 200-fold excess of unlabeled GDP. GEF-mediated exchange was initiated by adding 10 µM of the REM-CDC25 domain of SOS1 under identical conditions. Fluorescence changes were monitored with excitation at 355 nm and emission above 408 nm. For the nucleotide-binding assay, various concentrations of mant-dGDP (ranging from 0.2 to 0.8 µM) were mixed with 0.2 µM of nucleotide-free protein. The observed rate constants (kobs) were determined by fitting the curves to a single-exponential model using OriginLab 9.80 software. Association rate constants (k_on_) were calculated from linear plots of kobs versus nucleotide concentration. Dissociation rate constants (k_off_) were determined by displacement with an excess of unlabeled nucleotide. Equilibrium dissociation constants (K_d_) were calculated as the ratio of k_off_ to k_on_. The reported values represent the mean ± SD of three technical replicates.

### 2.5. GTP Hydrolysis Measurements

The intrinsic and GAP-stimulated GTP hydrolysis rates of RIT1 and HRAS were measured using fluorescence-based assays on a Fluoromax-4 spectrofluorometer (Horiba Scientific, Kyoto, Japan). For the intrinsic measurements, purified proteins were preloaded with 0.2 µM TAMRA-labeled GTP (TAMRA-GTP) and then diluted to a final volume of 200 µL in a quartz cuvette containing assay buffer consisting of 30 mM Tris-HCl (pH 7.5), 150 mM NaCl, 5 mM MgCl_2_, and 3 mM DTT. Reactions were carried out at 25 °C, and TAMRA fluorescence was recorded over time to monitor nucleotide hydrolysis. The excitation and emission wavelengths were set to 543 nm and 580 nm, respectively, with slit widths of 8 nm (excitation) and 10 nm (emission), respectively. For GAP-stimulated assays, 0.2 µM TAMRA-GTP-loaded protein was incubated with 1 µM of the catalytic domain of p120GAP under the same buffer and temperature conditions. Fluorescence decay was continuously recorded, and the k_obs_ values were obtained by fitting the decay traces to a single exponential function using OriginLab software.

### 2.6. Fluorescence Polarization

This assay was performed to determine the K_d_ values of direct protein–protein interactions. Measurements were carried out using a Fluoromax-4 fluorimeter (Horiba Scientific), which was operated in polarization mode. Purified RIT1 preloaded with mant-GppNHp was used at a fixed concentration in a total reaction volume of 200 µL. Binding reactions were conducted in quartz cuvettes at 25 °C in a buffer consisting of 30 mM Tris-HCl (pH 7.5), 50 mM NaCl, 5 mM MgCl_2_, and 3 mM DTT. Increasing concentrations of the binding partners were titrated into the solution, and the changes in polarization were recorded using an excitation wavelength of 360 nm (slit width: 8 nm) and an emission wavelength of 450 nm (slit width: 10 nm). Binding curves were analyzed in GraFit 5 by fitting the data to a quadratic ligand-binding equation to determine apparent K_d_ values. The error bars represent the fitting variation.

### 2.7. Lipid Strip Overlay Assay

We examined protein–lipid interactions using lipid strips (Echelon Biosciences, Salt Lake City, UT, USA), which contain immobilized phospholipids spotted onto a nitrocellulose membrane. The membranes were first blocked for one hour at room temperature in TBST (10 mM Tris-HCl, pH 8.0, 150 mM NaCl, and 0.1% Tween-20) supplemented with 3% fatty acid-free bovine serum albumin (BSA). The blocked membranes were then incubated for one hour at room temperature with purified GST-tagged proteins (full-length RIT1, RIT2, Cex constructs, or mutants), which were diluted in the same blocking buffer. After three washes with TBST containing 1% BSA to remove unbound protein, the membranes were incubated with an anti-GST primary antibody for one hour at room temperature. After three additional washes, the nitrocellulose membranes were incubated with the IRDye-conjugated secondary antibodies (LI-COR; see Section 2.10 for details) for one hour at room temperature. Subsequently, they were imaged using a LI-COR fluorescence imaging system. Lipid-binding profiles were compared across RIT1, RIT2, and their charge-reversal mutants to determine lipid specificity.

### 2.8. Liposome Preparation and Sedimentation Assay

For the assays, multilamellar vesicles were prepared using the following mixture of synthetic lipids dissolved in chloroform. The mixture was prepared according to the data obtained by lipid strip overlay assay. Each sample contained 100 µg of total lipid combined at the following weight ratios: 40% phosphatidylserine (PS), 25% phosphatidic acid (PA), 2% NBD-labeled phosphatidylethanolamine (NBD-PE), 12.5% phosphatidylethanolamine (PE), 10% phosphatidylcholine (PC), and a phosphoinositide (PI) mixture consisting of 2% PIP_3_, 2% PI(4,5)P_2_, 1.5% PI(3,5)P_2_, 1.5% PI(3,4)P_2_, 1.5% PI(3,4,5)P_3_, and 1% each of PI4 and PI5. The lipids were dried overnight under a laminar flow hood to form a thin film. The dried lipid film was hydrated in 20 mM HEPES-NaOH (pH 7.4), containing 100 mM NaCl. The mixture was homogenized by sonication using three cycles at 30% power. Each cycle consisted of 30 s of sonication followed by 2 min of cooling. There was a five-minute pause between cycles. To produce uniformly sized unilamellar vesicles, the suspension was extruded 21 times through a 100 nm polycarbonate membrane using an Avanti mini-extruder. Unlike the qualitative PIP strips, these synthetic liposomes form true lipid bilayers (approximately 100 nm in diameter), which are specifically formulated to resemble the charge density and lipid composition of the inner leaflet of a physiological plasma membrane biomimetically. For sedimentation assays, purified proteins were incubated with the prepared liposomes for 30 min on ice. The samples were then centrifuged at 20,000× *g* for 30 min at 4 °C. The input samples, consisting of the total protein amount before incubation with the liposomes, the supernatant containing the unbound proteins, and the pellet fraction containing the liposome-bound proteins, were collected separately and analyzed by SDS-PAGE followed by immunoblotting.

### 2.9. Cell Culture and Transfection

HEK293T cells were seeded in 10 cm culture dishes and maintained in Dulbecco’s Modified Eagle Medium (DMEM; Thermo Fisher Scientific, Cambridge, UK), which was supplemented with 10% fetal bovine serum (FBS) and 1% penicillin/streptomycin. The cells were then incubated at 37 °C in a humidified atmosphere containing 5% CO_2_. Fresh medium was added 12 h before transfection. When the cells reached approximately 70% confluence, they were transfected with 10 µg of plasmid DNA using TurboFect transfection reagent (Thermo Fisher Scientific) in 10 cm dishes. The transfection mixture was pre-incubated in serum-free DMEM for 20 min at room temperature before being added dropwise to the plates. Twenty-four hours later, the cells were washed with ice-cold phosphate-buffered saline (PBS) and lysed on ice for 10 min in a lysis buffer containing 50 mM Tris-HCl (pH 7.5), 5 mM MgCl_2_, 100 mM NaCl, 1% Igepal CA-630, 10% glycerol, 20 mM β-glycerophosphate, 1 mM sodium orthovanadate, and one EDTA-free protease inhibitor tablet per 50 mL. The lysates were cleared by centrifugation at 12,000× *g* for 10 min at 4 °C. The resulting supernatants were collected for downstream analysis. Immunoblot band intensities were quantified using Image Studio Lite, version 5.2, and normalized to the corresponding HA-RIT1, γ-tubulin, or GAPDH expression and loading controls, as needed. Quantitative analyses of p-ERK1/2, p-AKT (Thr308), and p-JNK1/2 were performed using data from three independent biological experiments.

### 2.10. Antibodies and Immunoblotting

Western blotting was performed to evaluate the expression and phosphorylation state of proteins in HEK293T cells following transfection. The lysates were mixed with Laemmli sample buffer, resolved on 12.5% SDS-polyacrylamide gels, and transferred to PVDF membranes via wet transfer at 120 V for 60–80 min. The membranes were blocked for one hour at room temperature, then incubated overnight at 4 °C with gentle shaking and the following primary antibodies diluted 1:1000: anti-HA (Cell Signaling Technology [CST], Danvers, MA, USA, catalog #2367), anti-RIT1 (Abcam, Cambridge, UK, catalog #ab53720), anti-RIT2 (Thermo Fisher Scientific, catalog #PA1-25559), anti-6x-His (Thermo Fisher Scientific, catalog #MA5-33032), anti-phospho-ERK1/2 (Thr202/Tyr204, CST, catalog #4370), anti-total ERK1/2 (CST, catalog #4696), anti-phospho-AKT (Ser473, CST, catalog #4060), anti-phospho-AKT (Thr308, CST, catalog #2965), anti-total AKT (CST, catalog #2920), anti-phospho-JNK (CST, catalog #9251), anti-total JNK (CST, catalog #9252), anti-phospho-p38 MAPK (Thr180/Tyr182, CST, catalog #9211), anti-total p38 MAPK (CST, catalog #8690), anti-γ-tubulin (Sigma-Aldrich, St. Louis, MO, USA, catalog #T5326), and anti-GAPDH (CST, catalog #2118). GST-tagged proteins were detected using an in-house anti-GST antibody. Secondary detection was performed using IRDye 680RD- or 800CW-conjugated anti-rabbit (LI-COR Biosciences, Lincoln, NE, USA, catalog #926-68073 and 926-32213) and anti-mouse (LI-COR Biosciences, Lincoln, NE, USA, catalog #926-68072 and #926-32212) antibodies. Immunoreactive bands were visualized using the Odyssey Fc Imaging System (LI-COR Biosciences, Lincoln, NE, USA). Band intensities were quantified using Image Studio Lite version 5.2 and normalized to the corresponding loading controls.

## 3. Results and Discussion

### 3.1. RIT1 and RIT2 Share Classical Structural Features but Exhibit Distinct Regulatory Properties

**Figure 2:**
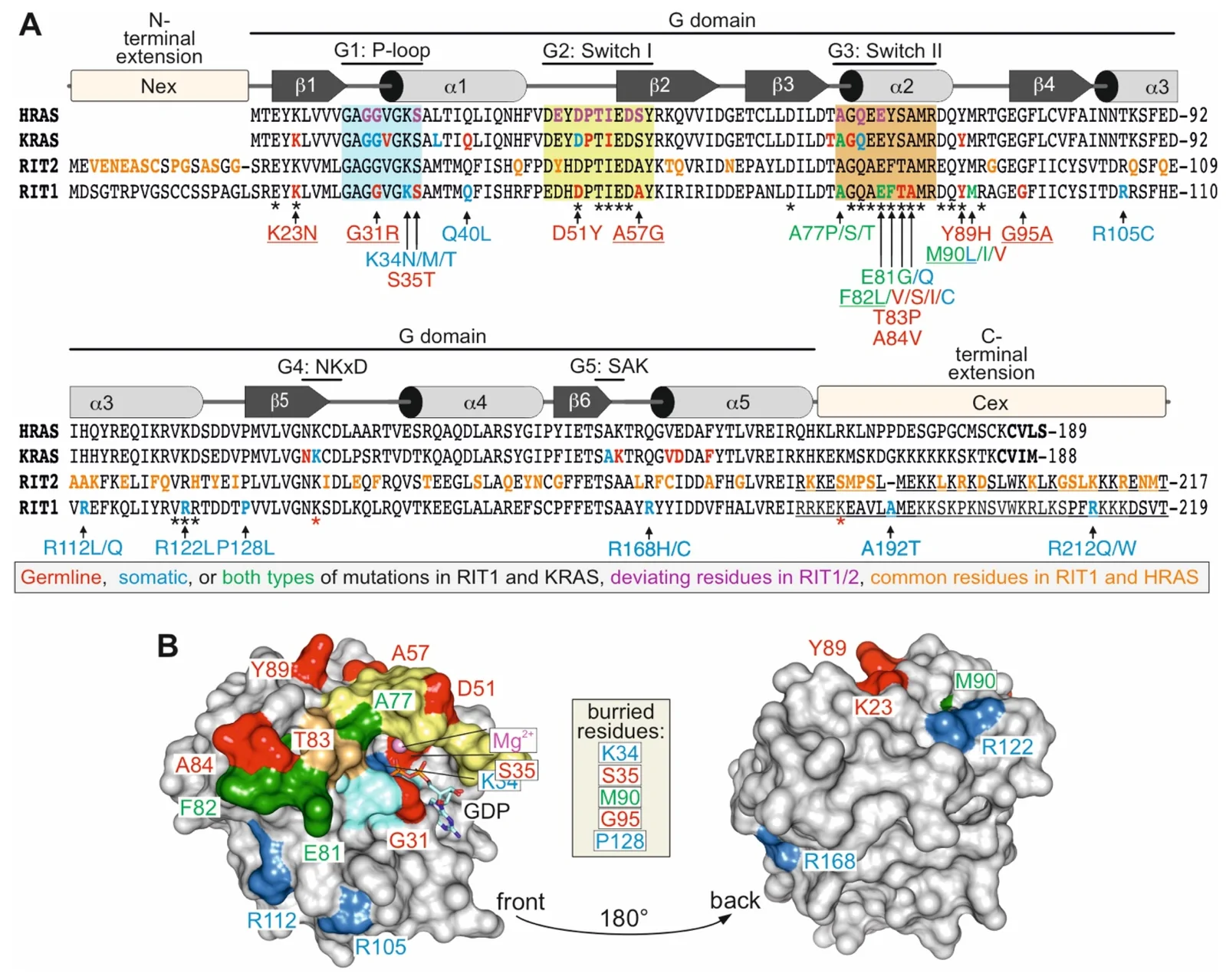
Distribution of disease-associated RIT1 mutations within conserved structural regions. (**A**) Schematic of the RIT1 G domain highlighting secondary structure elements (α-helices and β-strands) and five conserved motifs critical for guanine nucleotide binding and hydrolysis: G1 or P-loop (aquamarine), G2 or switch I (pale yellow), G3 or switch II (light orange), G4 (NKxD), and G5 (SAK). The positions of germline and somatic RIT1 mutations identified in individuals with malignancies or developmental disorders are mapped onto the linear domain representation. Secondary structure elements are derived from the RIT1•GDP structure (PDB ID: 4KLZ). In both KRAS and RIT1, germline mutations are shown in red, somatic mutations in blue, and mutations reported in both contexts are marked in green (adapted from Van et al., 2020 [9]). Orange residues in RIT2 indicate positions that differ from RIT1, while purple residues in HRAS denote positions that are conserved with RIT1 and implicated in intrinsic and GAP-stimulated GTP hydrolysis. Underlined mutations represent those analyzed experimentally in this study. Black asterisks highlight residues that interact with LZTR1 (adapted from Dharmaiah et al., 2025 [36]), and red asterisks mark the mapped ubiquitination sites (K135 and K187). Unlike HRAS (CVLS) and KRAS (CVIM), RIT1 and RIT2 lack a canonical C-terminal CAAX box yet retain membrane-binding capacity that has been associated with residues 203–214 (underlined). The amino acid sequences were retrieved from the UniProt database and aligned using ClustalW in BioEdit software. (**B**) Surface representation of the refined RIT1•GDP structure (PDB ID: 4KLZ) illustrating the spatial distribution of disease-associated mutations. Images showing surface representations of protein molecules were created using the PyMOL molecular viewer. Mutations linked to malignancies are shown in blue, those associated with developmental disorders in red, and those reported in both conditions in green. Front and back views of the structure, rotated by 180 degrees, are presented. The P-loop (aquamarine), switch I (pale yellow), and switch II (light orange) are highlighted to show the clustering of mutations in these regions. Residues buried within the hydrophobic core are boxed in yellow. This figure was created using CorelDRAW 2020.

Significant progress in the study of small GTPases has provided valuable insights into their structural features, regulatory mechanisms, and functions in various cellular processes and diseases. These findings provide a valuable foundation for understanding the lesser-known RIT proteins. Like other RAS family members, RIT1 and RIT2 contain a conserved GDP/GTP-binding domain (G domain), which governs the transition between active and inactive states. The G domain comprises five conserved motifs (G1 to G5) that are essential for guanine nucleotide binding, hydrolysis, and interactions with regulatory proteins such as GEFs, GAPs, and effectors (Figure 2A). Structural differences between the GDP- and GTP-bound states are localized primarily to two dynamic regions: switch I (residues 48–58) and switch II (residues 77–86) (Figure 2A) [34]. Although the basic switching mechanism is universally conserved among RAS GTPases, subtle structural and biochemical adaptations within the G domain enable individual family members to interact with different regulators and effectors, resulting in diverse signaling outputs (Figure 1) [35]. The crystal structure of the RIT1 G domain reveals a fold and secondary structure architecture that is highly similar to that of classical RAS proteins (Figure 2A). These conserved features suggest that RIT1 and RIT2 share the core structural switching mechanism characteristic of the RAS family. However, as shown in later sections, RIT1 and RIT2 exhibit regulatory and biochemical properties that differ from those of classical RAS GTPases despite this conserved G domain architecture.

### 3.2. RIT1 Displays Distinct Intrinsic Nucleotide Exchange and GTP Hydrolysis Rates

Structural and sequence comparisons of RIT1 and RIT2 with members of the classical RAS family, such as HRAS and KRAS, revealed notable differences in the sequence of the conserved G domain motifs, especially G2, G3, and G5 (Figure 2). These deviations suggest that RIT1 and RIT2 may exhibit distinct biochemical behaviors, especially with regard to GDP/GTP exchange, GTP hydrolysis, and interactions with regulators and effectors. To investigate the functional implications of these variations, we examined the nucleotide-binding properties of RIT1 in direct comparison with HRAS, the prototypical RAS GTPase.

**Figure 3:**
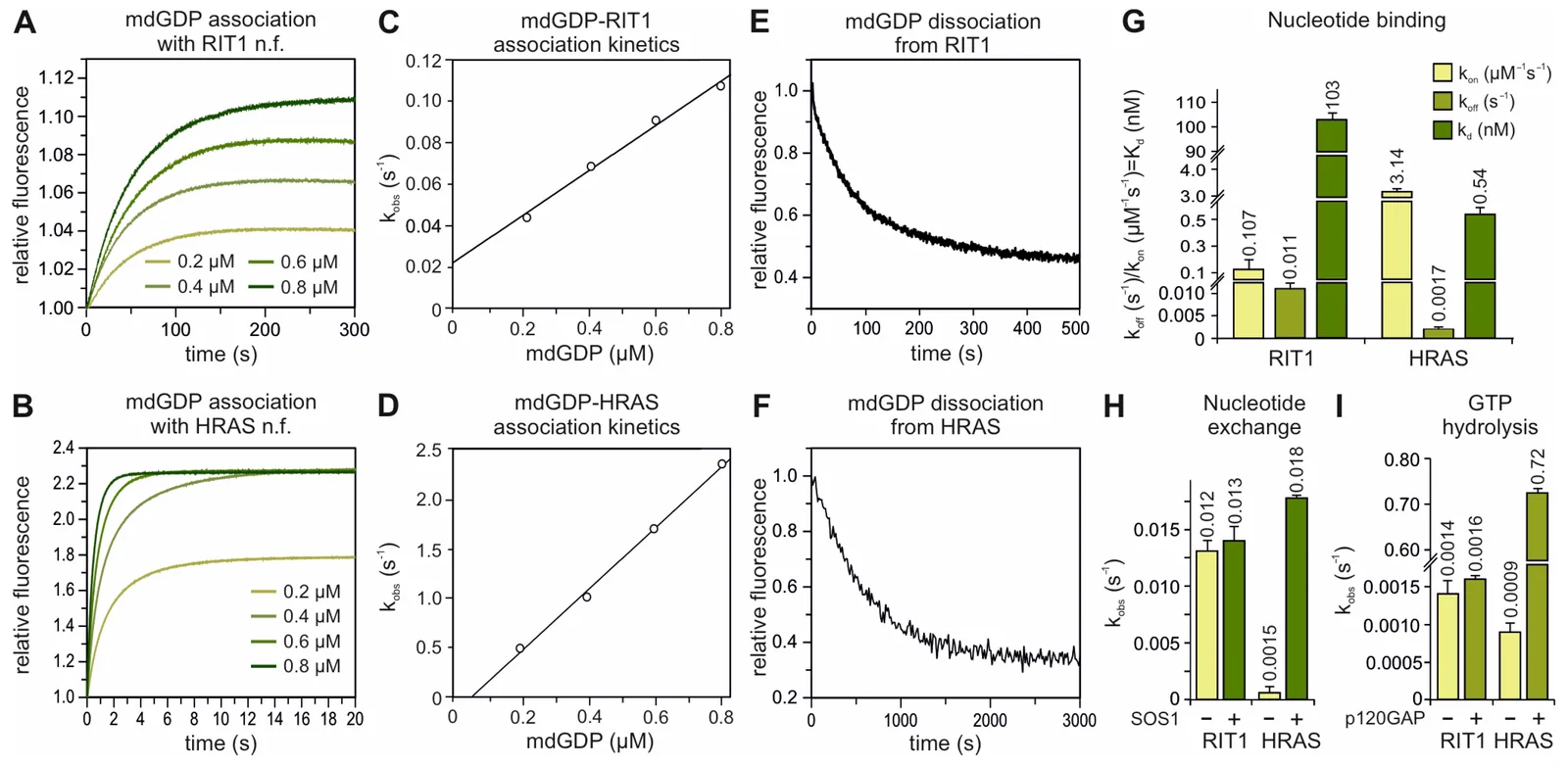
RIT1 GDP binding and GTP hydrolysis kinetics. (**A**,**B**) The association of increasing concentrations of fluorescent mant-dGDP with 0.2 µM nucleotide-free RIT1 (**A**) and HRAS (**B**) was measured using stopped-flow fluorimetry. (**C**,**D**) The association rate constants (k_on_) were determined by linear fitting of the kobs values plotted against the increasing mant-dGDP concentrations. (**E**,**F**) The intrinsic nucleotide dissociation from RIT1 and HRAS was monitored by measuring mant-dGDP release in the presence of excess unlabeled GDP. Dissociation rate constants (k_off_) were obtained by fitting the fluorescent traces to a single exponential function. (**G**) The calculated k_on_, k_off_, and dissociation constant (K_d_ = k_off_/k_on_) values for RIT1 and HRAS are shown as bar graphs. (**H**) Nucleotide exchange and (**I**) GTP hydrolysis rates were measured using 0.2 µM mant-dGDP–bound or TAMRA-GTP–loaded RIT1 and HRAS in the absence or presence of 10 µM REM-CDC25 domain of human SOS1 or 1 µM GAP domain of p120GAP. All kinetic parameters were derived by fitting the fluorescence traces to a single exponential function. Bar graphs represent mean ± SD from three technical replicates.

Using fluorescence spectroscopy with mant-dGDP, we found that RIT1 binds nucleotides with a substantially lower affinity than HRAS. As shown in Figure 3A–G, RIT1 exhibited a 30-fold lower k_on_, a 6.5-fold faster k_off_, and an overall 195-fold higher K_d_. These data suggest that the diminished nucleotide affinity of RIT1 is primarily due to its reduced association rate rather than enhanced nucleotide release. These results imply that efficient guanine nucleotide exchange of RIT1 may necessitate regulatory factors that differ from those identified for classical RAS proteins.

### 3.3. RIT1 Does Not Respond to the Classical RAS Regulators SOS1 and p120GAP Under Cell-Free Conditions

Next, we examined whether RIT1 responds to canonical RAS regulators. As expected, the catalytic core of SOS1 (SOS1-Cat), which consists of the CDC25 and REM domains, robustly stimulated nucleotide dissociation from HRAS but had no detectable effect on RIT1 (Figure 3H). These results suggest that RIT1 is not efficiently activated by SOS1 under the tested conditions. One possible structural explanation lies in the difference at residue F82 in RIT1, which corresponds to residue Y64 of HRAS. Y64 is a residue known to form a critical contact for SOS1. Previous studies have shown that mutating Y64 in HRAS abolishes SOS1-mediated exchange activity [35,37], supporting the idea that substitutions at this position may disrupt productive GEF engagement in RIT1. We then assessed GTP hydrolysis in the presence of the GAP domain of p120GAP. TAMRA-GTP hydrolysis by HRAS was strongly accelerated by the catalytic domain of p120GAP, whereas RIT1 remained unresponsive (Figure 3I). Most residues at the RAS-GAP interface are conserved in RIT1 and RIT2. However, key substitutions at H50 (Y32 in HRAS) in switch I and at A80 and F82 (E62 and Y64 in HRAS) in switch II may hinder GAP recognition and catalytic activation. Taken together, these results demonstrate that RIT1 does not respond to SOS1-mediated nucleotide exchange or p120GAP-stimulated GTP hydrolysis under the tested experimental conditions. These findings suggest that RIT1, and potentially RIT2, may rely on regulatory mechanisms distinct from those characterized for classical RAS proteins. However, the identity of the relevant physiological regulators and the broader regulatory framework governing RIT1 and RIT2 remain to be determined.

### 3.4. Disease-Associated RIT1 Mutations Cluster near Critical Regulatory Regions

Most disease-associated RIT1 mutations are located near the P-loop and switch II regions. This raises the possibility that the mutations may influence GAP-mediated regulation or effector interactions. We determined the structural localization and spatial orientation of these residues using the GDP-bound RIT1 crystal structure (PDB ID: 4KLZ). Since the available structure lacks several regions, additional modeling and refinement were performed to improve residue placement. To evaluate the potential impact of specific mutations on nucleotide binding or hydrolysis, we examined their proximity to the bound GDP. Three residues occupy key contact positions: G31 and K34 interact with the β-phosphate of GDP, and S35 coordinates the essential Mg^2+^ ion (Figure 2B). Substituting S35 with asparagine (S35N) may sterically disrupt Mg^2+^ coordination and decrease nucleotide affinity. This effect is similar to what has been observed in HRAS, where analogous mutations impair nucleotide binding and generate dominant-negative variants that alter RAS activation [32,38]. However, since most germline and somatic RIT1 mutations are located on the protein surface near the P-loop and switch II regions (Figure 2), they may preferentially affect interactions with GAPs or downstream effectors rather than directly impairing nucleotide binding [35]. Although the binding interfaces for effectors diverge substantially between RIT1 and HRAS, residues involved in GAP recognition and GTP hydrolysis remain relatively conserved (Figure 2A, magenta). These structural observations suggest that, although RIT1 retains several conserved residue alignments associated with the RAS-GAP interface, its unique sequence variations at these interfaces indicate a distinct regulatory interaction that requires biochemical testing.

### 3.5. Evaluation of Disease-Associated RIT1 Mutations as an Initial Screening Approach in HEK293T Overexpression Systems

To screen the initial functional consequences of disease-associated variants, we evaluated germline and somatic RIT1 mutants in transiently transfected HEK293T cells. In these overexpression experiments, the mutations produced only modest increases in MAPK, PI3K/AKT, and JNK pathway signaling above baseline levels. Previous studies have shown that germline mutations in HRAS, NRAS, KRAS, RRAS2 (TC21), and MRAS (RRAS1) typically lead to a mild gain-of-function phenotype [2,39,40]. Biochemical characterization of these RAS and RAS-related germline variants has categorized them into several mechanistic classes, most of which differ substantially from those of classical oncogenic RAS mutations. These biochemical alterations include enhanced intrinsic or GEF-mediated nucleotide exchange, impaired GAP-stimulated GTP hydrolysis, and increased effector interactions. Ultimately, these alterations result in a moderate gain-of-function phenotype.

The developmental relevance of RIT1 became evident with the discovery of germline mutations in individuals with Noonan syndrome, beyond its role in cancer [12,13,41,42]. This RASopathy is characterized by craniofacial abnormalities, congenital heart defects, and lymphatic dysfunction [11,13,43,44,45]. Individuals with RIT1 mutations display a higher frequency of cardiovascular and lymphatic anomalies compared to other Noonan subtypes [10,46,47]. Interestingly, the RIT1 P128L mutation was identified in a patient with lung adenocarcinoma and was associated with resistance to the KRAS G12C inhibitor adagrasib [48].

To investigate the functional implications of somatic and germline RIT1 mutations observed in cancer and developmental disorders, we mapped their structural locations and compared them with known KRAS mutations (Figure 2). Most hotspot mutations in both RIT1 and KRAS cluster near the G1 to G3 motifs, which are crucial for nucleotide binding, GTP hydrolysis, and interaction with regulatory proteins and effectors. This pattern is reminiscent of the functional alterations previously observed in NRAS germline variants [2,49]. Because RIT1 gain-of-function mutations have been associated with Noonan syndrome, a disorder involving dysregulated MAPK signaling, we examined their capacity to regulate MAPK and related signaling pathways [9,11,43,45].

**Figure 4:**
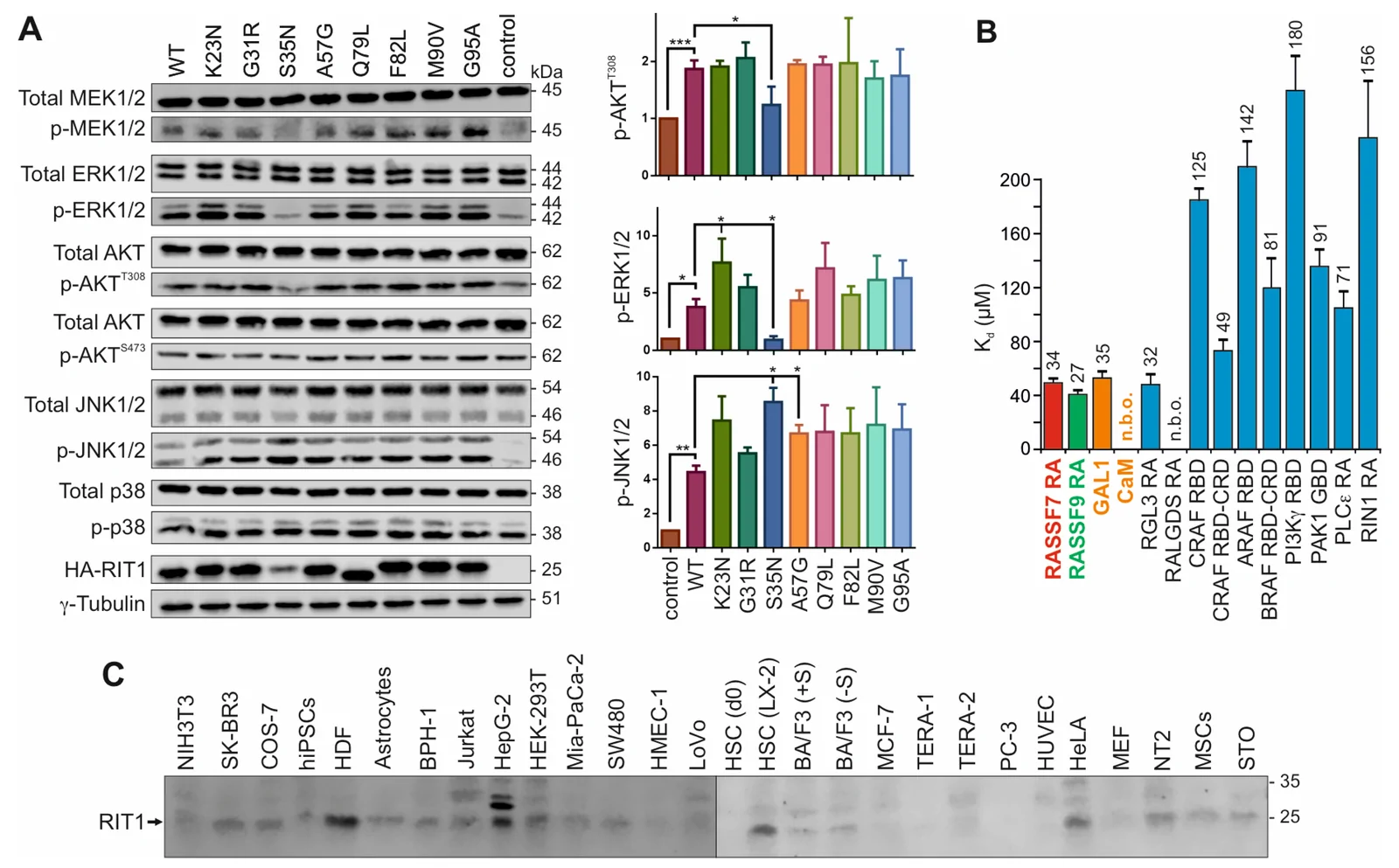
RIT1 signaling and effector binding characteristics. (**A**) HEK293T cells were transfected with RIT1 variants and analyzed via immunoblotting to detect MAPK, PI3K/AKT, and JNK signaling. Specific antibodies were used to analyze the phosphorylation and total protein levels of MEK1/2, ERK1/2, AKT (Ser473 and Thr308), JNK1/2, and p38. γ-tubulin served as a loading control. Immunoblot intensities of p-ERK1/2, p-AKT (Thr308), and p-JNK1/2 were quantified using Image Studio Lite version 5.2. The results were normalized to the corresponding HA-RIT1 expression and loading controls and represent three independent biological experiments. Statistical significance was determined using an unpaired, two-tailed Student’s *t*-test (* *p* < 0.05, ** *p* < 0.01, and *** *p* < 0.001). (**B**) Fluorescence polarization was used to determine the dissociation constants (Kd) of mGppNHp-bound RIT1 with various effector proteins. K_d_ values were obtained by fitting concentration-dependent binding curves to a quadratic ligand-binding equation. Error bars represent fitting variation. "n.b.o." denotes "no binding observed." Abbreviations: CaM: calmodulin; CRD: cysteine-rich domain; GAL1: galectin-1; GBD: GTPase-binding domain; RA: RAS-association domain; RBD: RAS-binding domain. (**C**) Immunoblot analysis of the endogenous RIT1 protein (Abcam #ab53720) in various human and mouse cell lines, including fibroblasts, stem cells, carcinoma cell lines, primary cells, and immortalized cell lines. All lanes were loaded with 20 µg of total cell lysate.

We overexpressed various RIT1 mutants, including the putative dominant-negative mutant S35N and the putative constitutively active mutant Q79L, in HEK293T cells. Then, we assessed the activation of MEK1/2-ERK1/2, JNK, and p38 signaling in comparison with wild-type RIT1 (Figure 4A). Unlike oncogenic KRAS mutations, which strongly activate MEK and ERK signaling [2,49], most RIT1 mutants showed only a mild or absent increase in their phosphorylation. This effect was restricted to a subset of variants. Overexpression of wild-type RIT1 alone increased the phosphorylation of MEK1/2, ERK1/2, AKT^T308^, and JNK1/2. However, expression of the mutants did not further enhance these signals. Consequently, the elevated expression levels inherent to this transient system may be a confounding factor. Therefore, caution should be exercised when interpreting downstream signaling outputs in the context of physiological processes.

The Q79L variant is considered constitutively active due to its impaired GTP hydrolysis and reduced GAP sensitivity [39]. However, expressing it in HEK293T cells did not result in stronger MAPK signaling than with wild-type RIT1. In contrast, a previous study in PC6 cells (a neuron-like pheochromocytoma cell line) reported elevated ERK1/2 and p38 phosphorylation in response to GFP-tagged RIT1 Q79L [16,50]. Previous data from HEK293T cells, however, showed similar GTP loading for Q79L and wild-type RIT1 under serum-starved conditions [51]. This discrepancy underscores the importance of interpreting these results within the framework of an overexpression model. High-level transient expression of wild-type RIT1 can easily saturate downstream signaling cascades, effectively masking mutant phenotypes. Additionally, HEK293T cells are a non-neuronal, non-developmental cell line that likely lacks the tissue-specific cofactors, regulators, and effectors necessary to manifest distinct pathogenic phenotypes. Furthermore, a recent study showed that RIT1 variants with mutations near the regulatory interface can evade LZTR1-mediated degradation. This raises the possibility that certain mutants may achieve sustained activation through reduced protein turnover [36]. Verifying protein levels and conducting dose–response analyses in future experiments could address this issue. Consequently, these experiments are a valuable initial screening method. However, they do not provide definitive physiological characterizations of disease-associated RIT1 variants. The stronger effects observed in PC6 cells may also reflect the presence of neuronal or context-specific cofactors in PC6 cells that are absent in HEK293T cells. These cofactors may be necessary for the full engagement of RIT1 signaling. Together, these findings suggest that RIT1 signaling properties differ from those of classical RAS proteins and depend on the cellular context and regulatory interactions [9]. It is important to note that these findings are based on transient overexpression in HEK293T cells and may not accurately reflect physiological signaling contexts. The observed signaling outputs may be influenced by factors specific to the cell type, expression levels, and the absence of relevant cofactors. Therefore, these results should be interpreted with caution and validated in more physiologically relevant systems.

### 3.6. The RIT1 S35N Mutant Exhibits Signaling Properties Consistent with a Potential Dominant-Negative Effect

A notable observation was made regarding the RIT1 S35N variant. This variant was found to exhibit reduced phosphorylation of ERK1/2 and AKT^T308^, but not JNK. Lower S35N protein levels were also detected (Figure 4A). The variant contains a mutation in a conserved residue within the P-loop (Figure 2A,B), which may favor a low nucleotide-binding state. This state exerts dominant-negative effects through altered interactions with regulatory factors [32,38]. Consistent with this interpretation, RIT1 S35N expression was associated with reduced MAPK and AKT^T308^ signaling, reflecting potential interference with endogenous RIT1 regulatory pathways. Interestingly, the degree of signaling inhibition was disproportionate to the reduced S35N protein levels. This finding supports the possibility of a dominant-negative effect rather than signaling inhibition solely reflecting lower expression. We hypothesize that S35N sequesters an unidentified, RIT-specific exchange factor, making it a useful tool for studying RIT1 regulatory mechanisms.

### 3.7. Assessment of Candidate RIT1 Effectors Reveals Weak and Atypical Interaction Profiles

Previous studies have proposed several proteins, including CRAF, PAR6, PAK1, SIN1, RALGDS, RGL3, and CaM, as potential RIT1 effectors [7,20,26,28,29,50,52]. These candidates span diverse structural classes and signaling pathways. In many cases, the proposed interactions were based on co-expression, in silico prediction, or analogy to classical RAS proteins rather than on direct biochemical evidence from quantitative binding assays.

To assess the specificity of the RIT1 effector, we examined the binding of selected candidates using purified proteins and fluorescence polarization assays (Figure 4B). Consistent with our previous report of micromolar binding of RASSF7 and RASSF9 to active-state RIT1 [53], we extended this analysis to other proteins. Since GAL1 and CaM have been reported to interact with HRAS and KRAS, modulating their membrane targeting [54], we included GAL1 as a positive control in our RIT1-binding experiments [7]. GAL1 exhibited measurable binding to active RIT1, whereas CaM showed no interaction under the tested conditions, suggesting that CaM binding may depend on calcium ions, membrane context, or other cofactors. Among the tested classical RAS effectors, RGL3 displayed measurable affinity. Binding of RIT1 to the isolated CRAF Ras-binding domain (RBD) was weak but improved approximately 2.5-fold when the cysteine-rich domain (CRD) was included in a tandem RBD-CRD construct. While these interactions were detectable, their affinities were substantially weaker than the strong nanomolar-range interactions characteristic of canonical RAS-effector complexes. These findings suggest that RIT1 may engage interaction partners that differ from those typically associated with classical RAS proteins.

Our data are consistent with the possibility that RIT1 and RIT2 interact with regulatory and signaling proteins different from those characterized for classical RAS proteins. Systematic proteomic and biochemical studies, particularly under conditions that better reflect membrane association or physiological signaling context, are necessary to identify RIT1 interaction partners with high confidence. Additionally, sequence differences between RIT1 and RIT2 (Figure 2) suggest that the two proteins may have different interaction selectivities, a possibility that should be explored in future comparative studies.

RIT1 has been associated with cellular proliferation, differentiation, and migration through signaling mechanisms that are not fully understood. Despite multiple reports proposing candidate RIT1-interacting proteins [16,20,51], direct effectors that clearly mediate its downstream functions are poorly defined. A comprehensive strategy to map the regulatory and effector landscape of RIT1 is lacking. Future studies using endogenous RIT1 in physiologically relevant systems with baseline expression levels (e.g., primary human dermal fibroblasts; see Figure 4C) and integrated biophysical, proteomic, and cellular approaches may help clarify the regulatory and interaction landscape of RIT1. These studies could employ CRISPR-mediated knockouts and endogenously tagged RIT1 variants to avoid the confounding effects of overexpression. Gel overlay assays are a particularly sensitive method for detecting direct protein–protein interactions and have been effective in identifying binding partners of small GTPases [55].

### 3.8. The C-Terminal Polybasic Extension Supports RIT1 and RIT2 Lipid Interactions with Anionic Lipids Under Cell-Free Conditions

RIT1 and RIT2 share approximately 73% sequence identity. However, most of their sequence differences are located outside the canonical G domain, within their N-terminal (Nex) and C-terminal (Cex) extensions (Figure 2). Similar terminal extensions are also found in other RAS family members [1]. Classical RAS proteins rely on C-terminal post-translational modifications, such as prenylation via a CAAX motif, for stable membrane association (Figure 2). In contrast, RIT1 and RIT2 lack this motif. Previous studies have suggested that their membrane localization is mediated by the Cex region [1,6,55,56].

**Figure 5:**
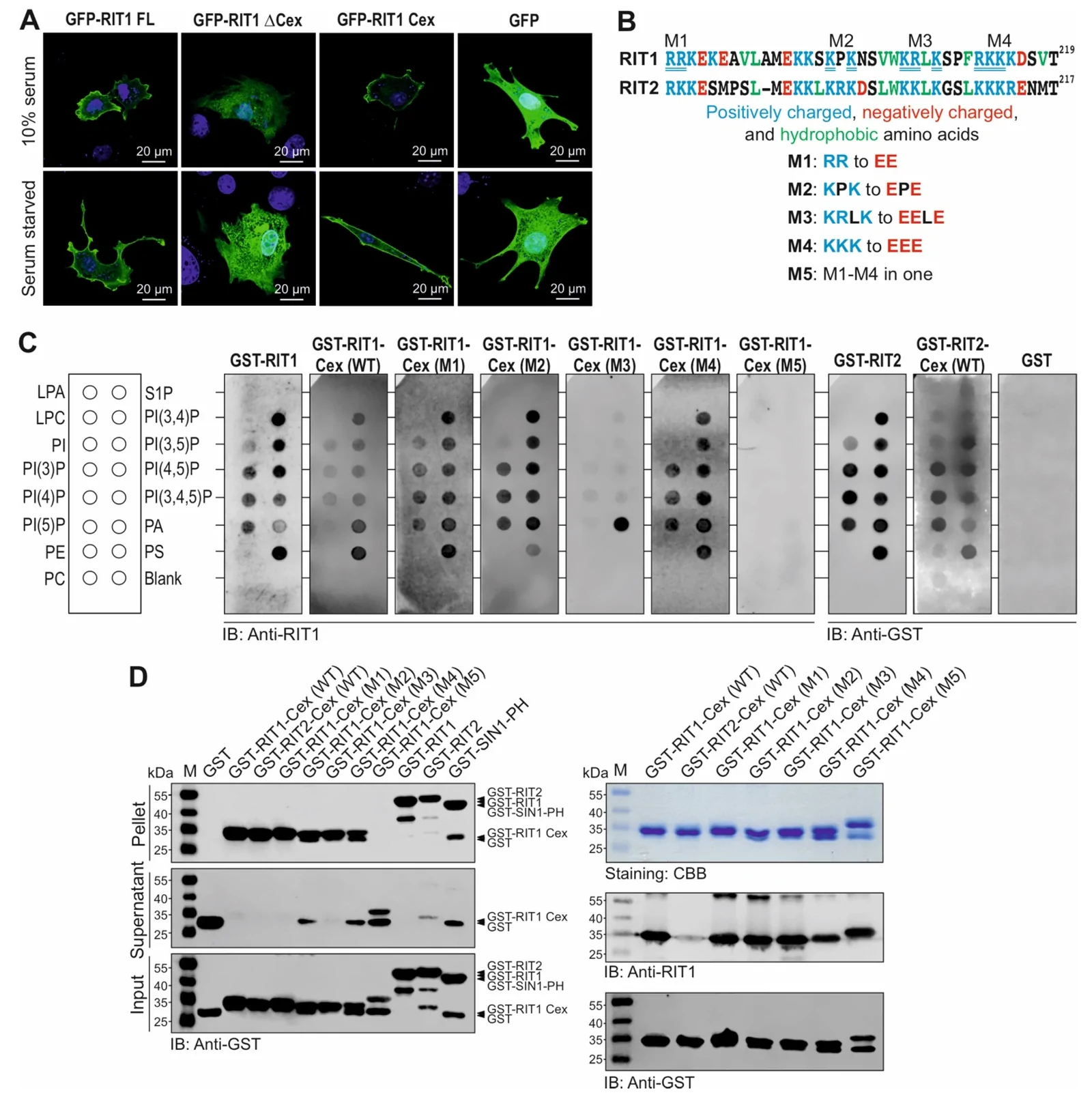
RIT1 and RIT2 lipid interaction mediated by their C-terminal extensions. (**A**) Confocal microscopy of NIH-3T3 fibroblasts expressing GFP-tagged RIT1 variants shows that full-length RIT1 (FL; residues 1–219) and the isolated C-terminal extension (Cex; residues 186–219) localize predominantly to the plasma membrane under both serum-starved and serum-stimulated conditions, whereas the Cex-deleted mutant (ΔCex; residues 1–185) remains largely cytoplasmic, similar to GFP alone. Nuclei were stained with DAPI (blue). Images were acquired using an LSM 510-Meta microscope (Zeiss, Jena, Germany); scale bars, 20 μm. (**B**) The Cex regions of RIT1 and RIT2 contain multiple positively charged motifs. An amino acid sequence alignment of the RIT1 Cex (residues 186–219) and the RIT2 Cex (residues 183–217) shows a similar distribution of positively charged residues (blue), negatively charged residues (red), and hydrophobic residues (green). Underlined residues in RIT1 indicate the Cex mutations (M1–M5) investigated in this study. M1–M4 are charge-reversal mutations of individual motifs, and M5 carries combined charge reversals of all four motifs. (**C**) Lipid-binding specificity was assessed using lipid strip overlay assays. Lipid strip nitrocellulose membranes were incubated with 200 ng/mL purified GST-tagged proteins and probed with RIT1 or GST antibodies; a RIT2 antibody was unavailable. GST-RIT1, GST-RIT2, and their Cex wild-type constructs, as well as the M1, M2, and M4 mutants, interacted with phosphoinositides (PIs), phosphatidylserine (PS), and phosphatidic acid (PA). In contrast, the M3 mutant showed substantially reduced binding to most tested lipids and retained detectable interaction primarily with PA, whereas the M5 mutant showed no detectable lipid interaction. GST alone showed no detectable binding. (**D**) Liposome sedimentation assays were performed using biomimetic liposomes of defined composition detailed in Section 2, selected based on the lipid strip results, to examine the lipid interactions of the full-length GST-RIT1, GST-RIT2, and wild-type or mutant Cex constructs. GST alone served as a negative control, and the GST-SIN1 PH domain served as a positive control. Full-length RIT1 and RIT2, along with the wild-type Cex constructs and most mutants (M1–M4), were predominantly recovered in the pellet fraction, whereas the M5 mutant showed no detectable liposome interaction. The right-hand panel shows a Coomassie- Brilliant Blue (CBB)-stained gel of purified Cex proteins (top), followed by immunoblots probed with anti-RIT1 (middle) and anti-GST (bottom), confirming protein purity and showing that the anti-RIT1 antibody detects RIT1 but not RIT2. Molecular weights are indicated in kilodaltons (kDa).

To determine whether the Cex region of RIT1 contributes to membrane targeting, we examined the subcellular localization of GFP-tagged RIT1 variants in NIH-3T3 fibroblasts. As shown in Figure 5A, the full-length RIT1 protein predominantly localized to the plasma membrane, while the ΔCex variant (residues 1–185) remained largely in the cytoplasm. However, the Cex region alone (residues 186–219) was sufficient to promote membrane localization. This finding suggests that the Cex region can support membrane association independently of the G domain.

Membrane-targeting peptides generally fall into two categories: (1) amphipathic α-helices enriched in hydrophobic residues and (2) unstructured polybasic regions [57,58]. The Cex regions of RIT1 and RIT2 belong to the second category. They consist of approximately 42% positively charged residues (lysine and arginine), which are distributed across a 35-amino-acid C-terminal segment (Figure 5B).

To test lipid specificity, we expressed and purified the full-length and C-terminal extensions of the GST-RIT1 and GST-RIT2 proteins, as well as their isolated Cex regions, and we performed lipid strip overlay and liposome sedimentation assays [52]. Full-length RIT1 and RIT2, their isolated Cex regions, and the RIT1 Cex mutants M1, M2, and M4 showed detectable interactions with PIs, PA, and PS but not with other membrane lipids under cell-free conditions (Figure 5C). This binding was abolished in GST-RIT1 Cex M5 and in GST-only controls. Mutant M5 contains charge-reversal substitutions across all four polybasic motifs. Conversely, mutant M3, which lacks K205, R206, and K208 (substituted with glutamate; see Figure 5B), retained PA binding but lost PI and PS recognition. These results suggest that these three basic residues are important for PI and PS interaction specificity. Together, our findings suggest that RIT1 and RIT2 interact with anionic lipid-enriched biomimetic liposomes under cell-free conditions through their C-terminal polybasic extensions.

Next, we examined the liposome-binding capabilities of RIT1, RIT2, their isolated Cex regions, and the RIT1 Cex mutants using a liposome sedimentation assay. This assay used biomimetic liposomes with a defined phospholipid composition, as guided by the lipid overlay results, and with an approximate diameter of 100 nm. GST fusion proteins were used for all experiments. GST alone served as a negative control, and the GST-SIN1 PH domain served as positive control. We assessed protein binding to liposomes by immunoblotting the pellet (bound) and supernatant (unbound) fractions. Figure 5D shows that full-length RIT1 and RIT2, as well as all Cex variants except Cex M5, were recovered in the pellet fraction, indicating that they have detectable liposome binding comparable to the positive control. The RIT1 Cex M5 mutant, which contains ten charge-reversal substitutions, failed to bind liposomes (Figure 5B,D), which is consistent with the reduction in positive charge that likely disrupts the electrostatic interaction with the membrane. In contrast, GST-RIT1 Cex M3 retained comparable liposome binding to the other Cex variants. This is consistent with its preserved PA binding (Figure 5C) and with the presence of sufficient basic residues remaining to mediate electrostatic interactions [59]. Previously reported molecular dynamics simulations are consistent with a model in which RIT1 associates with lipid bilayers through a cluster of 12 basic residues located between positions 203 and 214 (Figure 2A and Figure 5B) [60].

These findings support the idea that RIT1 and RIT2 use a distinct lipid interaction mechanism from the prenylation-dependent membrane targeting of classical RAS proteins. Many RAS regulatory interactions occur at or near cellular membranes [57], so the absence of a canonical CAAX motif in RIT1 and RIT2 raises questions about their membrane association mechanism. The identification of a polybasic C-terminal region that interacts with anionic lipids suggests a potential mechanism by which RIT1 and RIT2 associate with membrane compartments in a cell-free environment. These electrostatic interactions may also facilitate reversible membrane association under different cellular conditions. However, further investigation in cell-based and physiologically relevant systems is required to confirm this possibility.

The specific amino acid composition of the C-terminal polybasic region appears to be important for defining lipid selectivity, as suggested by the results of the lipid overlay assay. Full-length RIT1 and RIT2, their Cex regions, and the RIT1 Cex M1, M2, and M4 mutants interacted with PA, PS, and multiple PIs. The strong interaction with polyphosphorylated lipids such as PI(4,5)P_2_ and PI(3,4,5)P_3_, likely reflects their high local negative charge density. This density may engage the clustered basic residues of the polybasic region more effectively than simple electrostatic attraction alone [61,62]. The contrasting behaviors of the M3 and M5 mutants illustrate this dual mode of recognition. The complete loss of binding in the M5 mutant suggests that the overall positive charge is important for basal electrostatic attraction. In contrast, the selective loss of PI and PS binding in the M3 mutant indicates that the motif comprising K205, R206, and K208 strongly contributes to specific headgroup recognition. These results suggest that RIT1 membrane association is not solely governed by membrane charge density. Rather, the spatial positioning of these basic residues plays a role in the selective recognition of PI under cell-free conditions. Thus, RIT1 may be preferentially recruited to membrane microdomains that are enriched in polyphosphorylated PIs.

This membrane-targeting mechanism appears to depend on the polybasic Cex region and differs from the mechanism used by classical RAS isoforms. Unlike KRAS4B, which uses a dual prenylation and polybasic system for high-affinity membrane association [63], RIT proteins primarily depend on electrostatic interactions with anionic lipids. This makes their lipid affinity sensitive to local PI composition and the membrane environment. Previous studies have shown that RIT1 is enriched at the plasma membrane and within neurite extensions, which is consistent with a model in which this anionic lipid-responsive polybasic element permits transitions between membrane-associated and cytosolic states. Such dynamics may be necessary for neuronal differentiation and stress signaling. Our data from cell-free conditions, along with the identification of this lipid-sensitive C-terminal module, corroborate these observations. Together, they suggest that the positioning of RIT1 depends on the spatial distribution of anionic lipids within the plasma membrane.

This model also aligns with evidence that RIT1 undergoes post-translational regulation through the phosphorylation of serine 209 during mitosis. This modification modulates its interaction with mitotic checkpoint proteins. Phosphorylation within or near polybasic motifs is a well-established strategy for reducing local positive charge and weakening electrostatic interactions with anionic membranes [64]. Similarly, S209 phosphorylation may reduce the affinity of the RIT1 polybasic extension for anionic-lipid-rich membranes in a cell cycle-dependent manner. This could contribute to dynamic redistribution between membrane-bound and cytosolic states [65].

### 3.9. Galectin-3 and LZTR1, but Not Galectin-1, Reduce RIT1 and RIT2 Lipid Interactions in a Reconstituted Liposome System

The activity of the RAS family of small GTPases, including KRAS and HRAS as well as the atypical members RIT1 and RIT2, critically depends on their proper localization and nanoscale organization within the inner leaflet of the plasma membrane [8,66,67,68,69]. These proteins do not signal as freely diffusing monomers but rather as components of nanoclusters. The formation, stability, and signaling output of these nanoclusters are regulated by specific lipid interactions and accessory proteins [66,70,71,72,73].

Galectins, particularly GAL1 and galectin-3 (GAL3), have emerged as key scaffolding proteins that modulate HRAS and KRAS nanoclusters [67,72,73,74,75,76]. GAL3 interacts with KRAS through a hydrophobic pocket in its carbohydrate-recognition domain, which accommodates the farnesyl moiety and promotes nanocluster formation and sustained MAPK signaling [72,73,74,77]. GAL1 stabilizes HRAS nanoclusters independently of its carbohydrate-binding activity [67,75,76]. However, the regulatory effects of galectins on RIT1 and RIT2 remain unclear. Unlike classical RAS isoforms, RIT1 and RIT2 lack a CAAX motif and instead rely on a positively charged C-terminal extension for membrane engagement [8,56,60].

In parallel with galectin-mediated spatial organization, the cellular abundance of RAS family proteins is regulated by ubiquitin-dependent degradation [78,79]. LZTR1, a substrate adaptor for the CUL3 E3 ubiquitin ligase complex, regulates RAS proteostasis and specifically targets RIT1, MRAS, and KRAS for ubiquitination and proteasomal degradation [33,36,51,78,79,80,81]. Pathogenic mutations in either LZTR1 or RIT1 disrupt this regulatory interaction, resulting in increased RIT1 stability and enhanced MAPK signaling, which is a hallmark of Noonan syndrome and related developmental disorders [33,44,51,80,82,83,84]. Although the role of LZTR1 in regulating RIT1 abundance is well described, its influence on the membrane association properties of RIT1 and RIT2 remains unclear.

**Figure 6:**
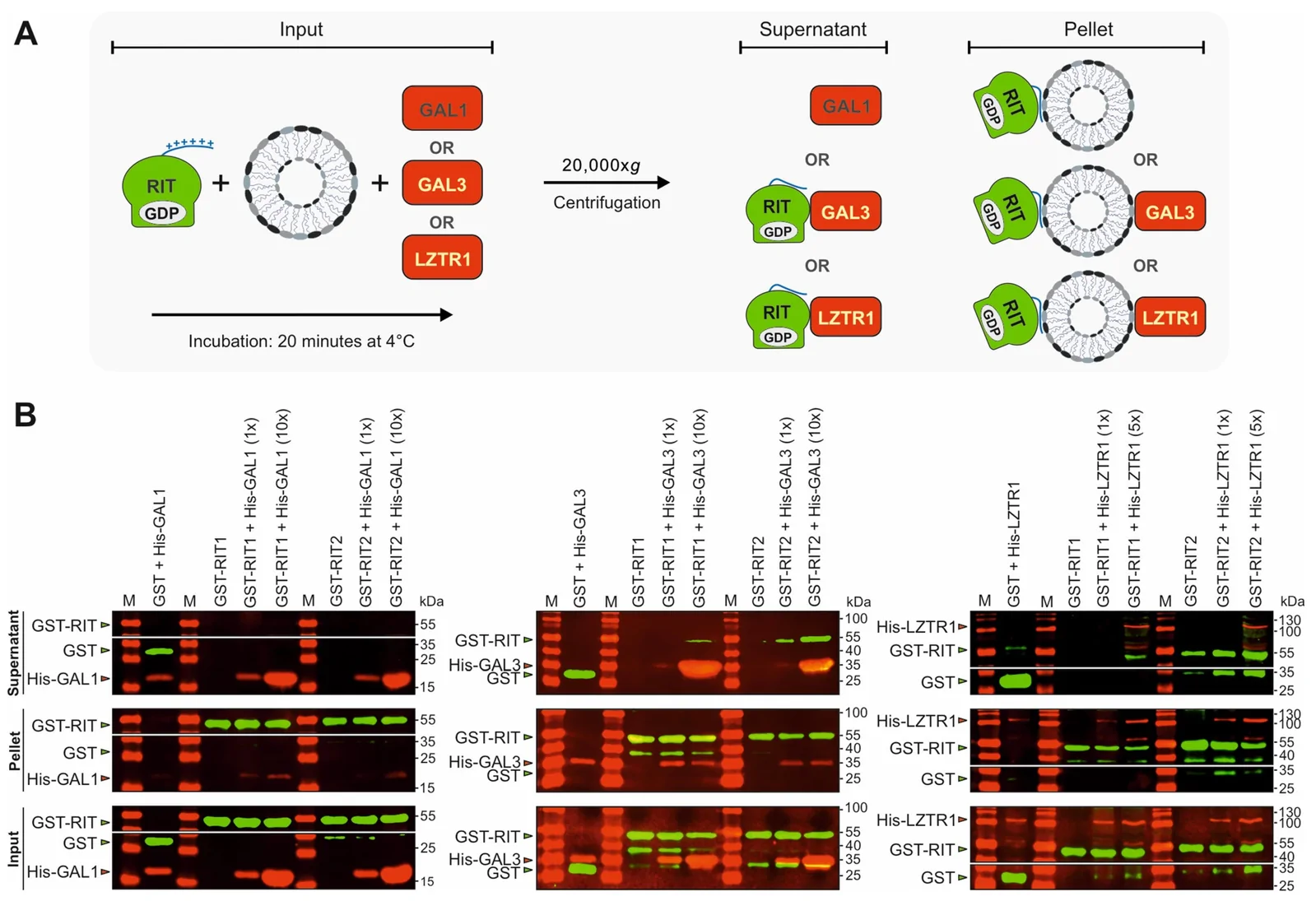
Effect of GAL3 and LZTR1 on the interaction of RIT1 and RIT2 with liposomes. (**A**) Schematic representation of the liposome sedimentation assay. Purified GDP-loaded GST-RIT1 or GST-RIT2 was incubated with liposomes of defined composition in the presence of GAL1, GAL3, or LZTR1, either at equimolar amounts or in excess. After incubation, the samples were centrifuged at 20,000× *g* to separate the liposome-associated proteins in the pellet fraction from the unbound proteins in the supernatant fraction. (**B**) Representative immunoblots of the input, pellet, and supernatant fractions from the liposome sedimentation assay. GST-RIT1 and GST-RIT2 were detected using anti-GST antibodies (green), and His-tagged GAL1, GAL3, and LZTR1 were detected using anti-His antibodies (red). In the presence of GAL1, GST-RIT1 and GST-RIT2 were predominantly detected in the pellet fraction (**left** panel). In contrast, increasing amounts of GAL3 (**middle** panel) or LZTR1 (**right** panel) showed representative qualitative increases in GST-RIT1 and GST-RIT2 signal within the supernatant fraction under these tested reconstituted conditions, without implying definitive quantitative comparison. GST alone showed no detectable liposome interaction.

To address these questions, we tested whether GAL1, GAL3, and LZTR1 affect the lipid interactions of RIT1 and RIT2 under cell-free conditions using a biomimetic liposome system. As detailed in Section 2, we conducted liposome sedimentation assays with purified GDP-loaded GST-RIT1 and GST-RIT2 proteins. These were then incubated with liposomes of a defined phospholipid composition, which was selected based on the lipid strip analysis. Analysis of the pellet (bound) and supernatant (unbound) fractions revealed representative qualitative decreases in the liposome association of GDP-loaded GST-RIT1 and GST-RIT2 in the presence of GAL3 and LZTR1, whereas no apparent change was observed with GAL1 under these tested reconstituted conditions (Figure 6). These results provide biochemical evidence that GAL3 and LZTR1 can affect RIT1/2 membrane interactions in a reconstituted system. They also suggest that GAL3 and LZTR1 may modulate the Cex-mediated lipid interaction mechanism depicted in Figure 5.

Representative immunoblots in Figure 6 show that GST-RIT1 and GST-RIT2 were predominantly detected in the pellet fraction in the presence of GAL1. Conversely, qualitative shifts toward the supernatant fraction were observed for GST-RIT1 and GST-RIT2 in the presence of GAL3 or LZTR1, particularly at higher protein ratios, reflecting qualitative differences in liposome association under these defined in vitro conditions. GST alone showed no detectable liposome association.

It is noteworthy that GAL3, but not GAL1, can reduce the association of RIT1 and RIT2 with liposomes, given that both galectins stabilize classical RAS nanoclusters [67,72,73,74,75,76,77]. Since RIT1 and RIT2 lack prenylation and primarily rely on a polybasic C-terminal extension for lipid association (Figure 5), GAL3 may act differently on RIT proteins than on classical RAS proteins. Possible mechanisms include competition for anionic lipids, alteration of local membrane charge, or steric interference that limits the access of the C-terminal basic region to the membrane bilayer [85]. However, it remains unresolved whether GAL3 exerts similar effects on endogenous proteins or redistributes RIT1/2 between plasma membrane subdomains.

Our data suggest that LZTR1 reduces the lipid association of GDP-loaded RIT1 and RIT2 in a reconstituted liposome system. Along with the previously identified RIT1 ubiquitination sites (K135 and K187), these results imply that LZTR1 may affect RIT1 interactions with the membrane in addition to its known function in proteasomal degradation [51,78]. Since K187 is located within the C-terminal extension (Figure 2), future studies must determine whether the physical presence of LZTR1 alters the topology or accessibility of the lipid-binding module.

These qualitative observations in liposome sedimentation for RIT1 and RIT2 in the presence of GAL3 and LZTR1 align with the lipid interaction mechanism as depicted in Figure 5. Polybasic regions primarily interact with liposomes through electrostatic interactions with negatively charged lipids, a reversible mechanism that is sensitive to local lipid composition and membrane potential [86,87]. The observed effects of GAL3 and LZTR1 suggest that RIT1/2 membrane interactions may be influenced by changes in the membrane environment or by protein–protein interactions. However, it remains to be determined whether similar regulatory effects occur under physiological cellular conditions.

From a broader regulatory perspective, our findings suggest that galectin-mediated spatial regulation and LZTR1-dependent proteostasis may intersect with the C-terminal polybasic region, which contributes to RIT1/2 membrane interactions. This intersection may be relevant in disease contexts such as Noonan syndrome, where RIT1 mutations and LZTR1 variants have been associated with altered MAPK pathway regulation [33,51,80,82,83,88].

Finally, we note that all assays were performed in a reconstituted system with purified proteins and synthetic liposomes. While this approach is well suited for establishing proof of principle, it does not fully replicate the complexity of cellular membranes or native nanocluster microdomains. Future studies should determine whether endogenous GAL3 and LZTR1 similarly influence the localization, membrane dynamics, and turnover of RIT1 and RIT2 in intact cells. Additional kinetic and structural analyses may help define how accessory proteins influence the balance between membrane-associated and cytosolic states. Despite these limitations, our observations provide qualitative cell-free evidence that GAL3 and LZTR1 can alter RIT1/2 liposome association under defined reconstituted conditions. Our results also identify these proteins as potential modulators of RIT membrane association.

## 4. Concluding Remarks and Future Directions

This study provides biochemical insights into the regulation, effector interactions, and lipid-binding properties of the small GTPases RIT1 and RIT2. Although RIT1 and RIT2 have a GDP/GTP-dependent molecular switch architecture similar to core RAS proteins, our cell-free assays revealed that RIT1 has distinct nucleotide-cycling properties. Under the cell-free conditions used here, RIT1 showed no detectable response to the tested SOS1 and p120GAP constructs. Furthermore, cell-based assays demonstrated that RIT1 mutations associated with disease, which cluster in the P-loop and switch II regions, had modest effects on the examined canonical MAPK and PI3K/AKT signaling pathways when overexpressed. Reconstituted liposome assays showed that the C-terminal polybasic extension (Cex) contributes to the membrane association of both RIT1 and RIT2 through electrostatic interactions with negatively charged lipids such as phosphatidylserine and phosphoinositides. Qualitative liposome sedimentation observations indicated that GAL3 and LZTR1, but not GAL1, led to apparent reductions in RIT1 and RIT2 liposome association. These representative data suggest that GAL3 and LZTR1 are candidate modulators of RIT membrane association in cell-free systems under the tested reconstituted conditions, though further cellular studies are required.

Based on these experimental findings, we propose a dual-layer regulatory hypothesis for RIT GTPases that integrates nucleotide switching with non-prenylated membrane association. First, since RIT1 did not respond to the SOS1 and p120GAP constructs tested under the cell-free conditions used here, intracellular nucleotide cycling may involve intrinsic mechanisms or unidentified RIT-specific regulatory proteins. Second, the distribution of RIT GTPases between the cytosol and membrane may be influenced by the positively charged Cex domain and its interaction with anionic membrane lipids. Accessory protein binding could alter this interaction, thereby influencing membrane association. Another speculative possibility is that post-translational modifications within Cex, such as phosphorylation at residue S209, could alter its interaction with anionic lipids. Therefore, we hypothesize that Cex modifications and accessory protein binding contribute to the regulation of RIT membrane association. These proposed mechanisms remain to be tested experimentally.

These conclusions should be interpreted in light of the limitations of the present study. The reconstituted liposome system does not replicate the molecular complexity, protein crowding, lipid organization, or dynamic lipid turnover of native cellular membranes. Additionally, HEK293T signaling analyses were performed under transient overexpression conditions, which may mask the subtle effects of RIT1 mutations. The limited availability of suitable antibodies also constrained the characterization of endogenous RIT2 at the cellular level. Furthermore, the physiological regulators and high-affinity effectors of RIT1 and RIT2 are not fully understood.

To address these questions and reduce artifacts caused by overexpression, future studies should use CRISPR-mediated endogenous tagging in cell lines with high RIT expression, such as those shown in Figure 4C. Furthermore, quantitative biophysical approaches such as surface plasmon resonance (SPR) combined with atomistic molecular dynamics simulations could help determine the binding kinetics and residence times of the Cex region on biomimetic membranes. Finally, proximity-dependent biotinylation approaches, such as BioID/TurboID, could help identify authentic physiological interaction partners and clarify how RIT GTPase networks contribute to RASopathies and malignancies in human induced pluripotent stem cell (iPSC)-derived neurons or relevant developmental models.

## Data Availability

All data generated or analyzed during this study are included in this published article. Additional information is available from the corresponding author upon reasonable request.

## Abbreviations

The following abbreviations are used in this manuscript:

AKT	Protein kinase B
CAAX	Cysteine–aliphatic–aliphatic–X motif
CaM	Calmodulin
CBB	Coomassie Brilliant Blue
Cex	C-terminal extension
CRD	Cysteine-rich domain
DMEM	Dulbecco’s Modified Eagle Medium
DTT	Dithiothreitol
EGF	Epidermal growth factor
EPAC1	Exchange protein directly activated by cAMP 1
FBS	Fetal bovine serum
GAL1	Galectin-1
GAL3	Galectin-3
GAP	GTPase-activating protein
GBD	GTPase-binding domain
GDP	Guanosine diphosphate
GEF	Guanine nucleotide exchange factor
GTP	Guanosine triphosphate
HA	Hemagglutinin
HDF	Human dermal fibroblasts
HRAS	Harvey rat sarcoma viral oncogene homolog
IGF1	Insulin-like growth factor 1
JNK	c-Jun N-terminal kinase
K_d_	Dissociation constant
k_obs_	Observed rate constant
k_on_	Association rate constant
LPA	Lysophosphatidic acid
LPC	Lysophosphocholine
LZTR1	Leucine Zipper-like transcription regulator 1
MAPK	Mitogen-activated protein kinase
Mant-dGDP	2′−O−(N−Methylanthraniloyl)−2′−desoxyguanosin−5′−diphosphate
Mant-GppNHp	Mant-2′−(3′)−N−Methylanthraniloyl−guanosin−5′−(β,γ−imido)triphosphate
MSK1	Mitogen- and stress-activated kinase
Nex	N-terminal extension
NGF	Nerve growth factor
PA	Phosphatidic acid
PACAP38	Pituitary adenylate cyclase-activating polypeptide 38
PAK1	p21-activated kinase 1
PAR6	Partitioning defective protein 6
PBS	Phosphate-buffered saline
PC	Phosphatidylcholine
PE	Phosphatidylethanolamine
PDEδ	Phosphodiesterase δ
PI	Phosphoinositide or phosphatidylinositol
PI3K	Phosphoinositide 3-kinase
PIP	Phosphatidylinositol phosphate
PKA	Protein kinase A
PS	Phosphatidylserine
PVDF	Polyvinylidene difluoride
RA	RAS-association domain
RAS	Rat sarcoma
RBD	RAS-binding domain
RGL3	RAL guanine nucleotide dissociation stimulator-like 3
RIT1	RAS-like without CAAX 1
RIT2	RAS-like without CAAX 2
ROSS	RAS of small size
S1P	Sphingosine 1 phosphate
SDS-PAGE	Sodium dodecyl sulfate–polyacrylamide gel electrophoresis
SIN1	Stress-activated protein kinase-interacting protein 1
SOS1	Son of sevenless homolog 1
SPR	Surface plasmon resonance
TAMRA	Tetramethylrhodamine
WT	Wild type
